# Comparison of K-Means and Hierarchical Clustering Methods for Buffalo Milk Production Data

**DOI:** 10.3390/ani15223246

**Published:** 2025-11-09

**Authors:** Lucia Trapanese, Giovanna Bifulco, Matteo Santinello, Nicola Pasquino, Giuseppe Campanile, Angela Salzano

**Affiliations:** 1 Department of Veterinary Medicine and Animal Production, University of Naples Federico II, 80137 Naples, Italy; lucia.trapanese2@unina.it (L.T.); matteo.santinello@unina.it (M.S.); giucampa@unina.it (G.C.); angela.salzano@unina.it (A.S.); 2 Department of Electrical Engineering and Information Technologies, University of Naples Federico II, 80125 Naples, Italy; nicola.pasquino@unina.it

**Keywords:** machine learning, buffalo, clustering, K-means, unsupervised analysis

## Abstract

**Simple Summary:**

In Italy, buffalo farming has gained increasing importance in recent years, with a steady rise in the number of animals reared. To optimize herd management and extract meaningful insights from available datasets, the use of Machine Learning techniques has become increasingly valuable. Among these, unsupervised learning provides a relatively straightforward and efficient approach for analyzing unlabeled data. In this study, we applied two unsupervised algorithms to routine buffalo production data to assess their performance and to evaluate their potential with the aim of improving herd management practices. Our findings suggest that, despite certain limitations, clustering analysis can offer substantial advantages in understanding herd structure, consistency and management.

**Abstract:**

This study investigated the use of K-means and hierarchical clustering, to group Italian Mediterranean buffalo using routinely collected test-day records. The analysis was first conducted on a combined dataset comprising three buffalo herds and subsequently on each herd individually. The main objective was to determine whether data-driven groupings could be implemented to support improvements in general herd management strategies. Results indicated that K-means consistently outperformed hierarchical clustering across all datasets, as reflected by average silhouette scores (0.17–0.18 vs. 0.10–0.12 for K-means and hierarchical, respectively), favorable Davies–Bouldin Index (DBI; 2.05–2.16 vs. 2.11–2.5 for K-means and hierarchical, respectively) and Calinski–Harabasz Index values (CHI; 1034–3877 vs. 729–2109 for K-means and hierarchical, respectively). K-means identified two clusters in the combined dataset and in two of the three herds, while three clusters were identified in the remaining herd. Cluster composition analysis revealed that days in milk and milk yield were the main discriminating factors when two clusters were formed. When three clusters emerged, K-means also identified a subgroup of animals that differed from the others in both age and lactation stage. These findings were supported by the analysis of variance (ANOVA), which showed statistically significant differences among most of the evaluated variables.

## 1. Introduction

The livestock sector is undergoing a profound transformation aimed at improving production efficiency while addressing environmental and economic sustainability challenges. As highlighted by Gong et al. [1], the growing global demand for animal-based products must be reconciled with the need to ensure animal welfare and the reduction in the environmental footprint, concerns increasingly prioritized by both researchers and consumers. In this regard, Peyraud et al. [2] observed that consumer preferences are progressively shaped by factors such as product quality, animal husbandry conditions and the nutritional value of processed animal products. Meeting these multifaceted expectations requires a shift from traditional livestock management toward the integration of advanced technologies and data-driven approaches. This transition is embodied in the concept of Precision Livestock Farming (PLF), which involves the adoption of real-time monitoring systems for animals, their products and their surrounding environment. The goal is to provide farmers with objective, timely and actionable data to enhance farm management practices [3].

Among emerging technologies, Machine Learning (ML) has gained prominence as a powerful tool for pattern recognition and decision support. In particular, ML techniques are well suited to handle large, complex, non-linear and noisy datasets, typical of livestock systems [4]. The ML algorithms are generally categorized into supervised and unsupervised, depending on whether the input data contains labeled outcomes [5]. Supervised algorithms are typically applied to classify events or predict values, such as nitrogen emissions [6] or reproductive efficiency [7]. Whereas unsupervised techniques are valuable for exploring data structures and detecting latent patterns. Indeed, Tremblay et al. [8] combined Principal Component Analysis (PCA) with the K-means algorithm to cluster cows affected by Poor Metabolic Adaptation Syndrome (PMAS). The five resulting clusters were classified into low, intermediate, or high PMAS categories based on their agreement with expected indicators and characteristics. Similarly, Franceschini et al. [9] assessed the overall health status of dairy cattle using large-scale milk-recording data, identifying four closely related clusters and one distinct group characterized by higher somatic cell score (SCS).

Although ML applications have become increasingly common in dairy cattle research, their use in other livestock species remains limited. Among these, dairy buffaloes represent a major component of the global milk market; however, the potential of ML in this field remains largely underexplored [10]. Buffalo farmers collect extensive routine data that, while already valuable for herd management and genetic improvement, still hold untapped potential for further optimization. In this context, unsupervised ML approaches can effectively exploit such information [11]. To address this gap, the present study has investigated the application of unsupervised ML techniques to routine test-day recording from three dairy buffalo farms. Specifically, two clustering algorithms, namely the K-means and hierarchical clustering, were applied to identify natural groupings of animals based on their production and physiological characteristics. The aim was to evaluate the potential of unsupervised learning to enhance herd management by grouping animals with similar physiological and productive profiles.

## 2. Materials and Methods

### 2.1. Data Description and Preprocessing

Animal welfare approval was not required for this study because all data were obtained from pre-existing databases. The dataset consisted of routinely collected records from three commercial dairy buffalo farms located in southern Italy. Animal characteristics and production data have been collected from the national test-day recording system managed by the Italian Breeders’ Association (AIA) and covered the period between 2013 and 2016. Only animals with fewer than six lactations were included. Before the analysis, a data-cleaning process was performed to ensure uniformity and reliability of the dataset. In this context, missing values, zeros and outliers were removed. Outlier detection thresholds were defined based on literature references and biological plausibility to ensure consistency [12]. The dataset comprised herds (*h*) containing a variable number of buffaloes *m_h_*. For each animal (*i*) within herd *h*, multiple measurements *p _h, i, j_* were collected over time, with sampling frequencies varying according to the farm’s recording schedule (one test-day per month). Overall, the dataset included 1.342 Italian Mediterranean buffaloes and a total of 16.702 test-day records. Data included information on:

- Animal and milk yield characteristics: parity order, daily milk yield, days in milk (DIM), age, lactation length and total lactation milk yield;
- Reproduction information: age at first calving (month);
- Milk quality: fat, protein, lactose, SCS and the fat/protein ratio.

The selection of the variables was aimed at capturing the multifaceted aspects of buffalo performance and management. Most of these variables were available from the routine recording system and were selected to represent three main areas of interest: animal and production traits, reproductive performance and milk quality. In addition, a correlation matrix including all variables in the dataset was computed to assess inter-variable relationships (Supplementary Materials Figure S1). The SCS was calculated from the Somatic Cell Count (SCC) using the formula proposed by Ali and Shook [13]:

$$SCS=3+\log2(\frac{SCC}{10^{5}})$$

**Figure 1:**
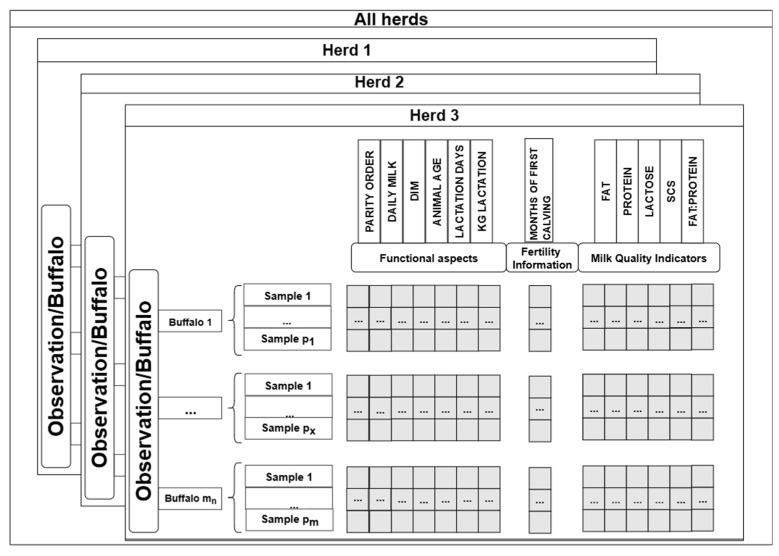
Schematic representation of the dataset structure used for clustering analysis.

All variables were standardized using z-score normalization to ensure comparability across features and to improve the efficiency and accuracy of the clustering process [9]. The structure of the final dataset was illustrated in Figure 1.

### 2.2. Cluster Analysis

The analysis was performed using R software (version 4.5.1; https://www.r-project.org/ accessed on 14 January 2025). A first analysis was initially carried out on the combined dataset (made of three different herds) to identify the most suitable clustering technique. Based on this preliminary approach, the best-performing algorithm was subsequently applied to each of the three herd-specific subsets to validate its effectiveness under different farm conditions. Cluster analysis was carried out using two unsupervised algorithms: K-means and hierarchical clustering. These methods were selected due to their previous application in cattle studies [14,15] and their compatibility with the structure of the dataset. Other unsupervised algorithms, such as Density-Based Spatial Clustering of Applications with Noise (DBSCAN) and Self-Organizing Maps (SOM), were also evaluated. However, DBSCAN, being density-based, was excluded since it was more suitable for anomaly detection [16]. Moreover, even if the SOM method is useful for time-series analysis, it requires a large number of continuous observations and for this reason it was not applied in our case, as the dataset included few test-day records per animal within one lactation [17]. For hierarchical clustering a Ward’s linkage method was employed, following recommendations from previous research [14]. A key challenge in such an unsupervised ML approach consists of determining the optimal number of clusters, which is not known a priori. To address this, the “NbClust” function from the NbClust R package (version 3.0.1) [18] was used. This function evaluates 30 different clustering indices to identify the most appropriate number of clusters for both algorithms. Clustering performance was assessed using four internal validation metrics: the average silhouette coefficient, the Davies–Bouldin Index (DBI), the Calinski–Harabasz Index (CHI), and the Dunn Index [19].

The silhouette coefficient evaluated how well each observation fits within its assigned cluster compared to other clusters. For each observation i, the silhouette coefficient is defined as:

$$s(i)=\frac{b(i)-a(i)}{max(a(i),b(i))}$$

where a(i) represents the average distance between a sample and all other points within the same cluster (a measure of intra-cluster cohesion), and b(i) represents the minimum average distance between a sample and all points in other clusters (a measure of inter-cluster separation). Specifically, a(i) is defined as:

$$a\left(i\right)=\frac{1}{\left|C_{i}\right|-1}\sum_{\begin{array}{c} x_{j}\;\in \;C_{i}\\ j\neq i \end{array}}d(x_{i},x_{j})$$

b(i) is defined as:

$$b\left(i\right)=\underset{j\neq i}{\min}\left(\frac{1}{\left|C_{j}\right|}\sum_{x_{j}\in C_{j}}d\left(x_{i},x_{j}\right)\right)$$

where $d(x_{i},x_{j})$ is the Euclidean distance between points $x_{i}$ and $x_{j}$ and $\left|C_{i}\right|$ is the total number of points in cluster i. The average silhouette coefficient (AVG_SIL) was then computed for each cluster as:

$$AVG\_SIL=\frac{1}{N}\sum_{i=1}^{N}\frac{b\left(i\right)-a(i)}{max\{a\left(i\right),b\left(i\right)\}},$$

where N is the total number of observations (within a cluster or across all clusters); a(*i*) is the average distance between observation *i* and all other points in the same cluster, while b(*i*) is the minimum average distance between *i* and points in any other cluster (i.e., the nearest neighboring cluster). The silhouette score ranges from −1 to 1. Values close to 1 indicate that the observation is well clustered, while negative values imply that the observation may be misclassified. The Davies–Bouldin Index (DBI) quantifies the compactness and separation of clusters, with lower values reflecting superior clustering performance. The DBI was computed following Davies et al. [20]:

$$DBI=\frac{1}{M}\sum_{j=1}^{M}\underset{j\neq k}{\max}R_{j,k},$$

where M is the total number of clusters, and R_j,k_ is the similarity between clusters j and k, typically defined as the ratio of within-cluster dispersion to between-cluster distance.

The CHI is determined by the ratio of inter-cluster dispersion to intra-cluster dispersion across all clusters:

$$CHI=\frac{SSB}{SSW}\cdot \frac{\left(N-M\right)}{\left(M-1\right)}$$

where N is the total number of observations of the dataset, SSB is the between-cluster sum of squares and SSW the within-cluster sum of squares, computed as

$$SSB=\sum_{i=1}^{M}\left|C_{i}\right|\cdot d(c_{i},c)$$

where $c_{i}$ is the centroid of cluster i and c the overall dataset centroid.

The within-cluster sum of squares (SSW) is calculated as:

$${SSW}_{K}=\sum_{i=1}^{\left|C_{i}\right|}d(x_{i},c_{k})$$

which measures the intra-cluster dispersion for each cluster, leading to:

$$SSW=\sum_{i=1}^{M}{SSW}_{i}$$

Finally, the Dunn Index measures the relationship between the minimum separation among clusters and the maximum dispersion within clusters. Higher Dunn Index values correspond to better clustering performance. It is defined as:

$$Dunn=\frac{{min}_{1\leq i<j\leq M}\delta (C_{i},C_{j})}{{max}_{1\leq l\leq M}∆(C_{l})}$$

where $\delta (C_{i},C_{j})$ is the distance between clusters *i* and *j* and $∆(C_{l})$ is the diameter of cluster *l*, defined as the maximum distance between any two points within the same cluster.

**Figure 2:**
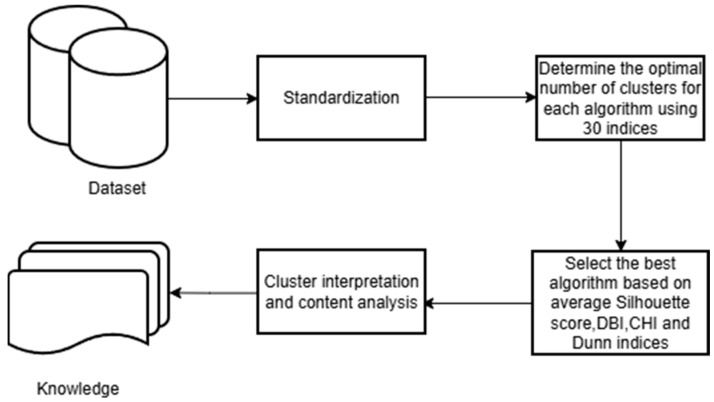
Overview of the analytical workflow adopted in the study. DBI = Davies–Bouldin Index, CHI = Calinski–Harabasz Index.

Figure 2 summarizes the workflow of the study. Starting from the dataset, the first step involved applying z-score standardization to all variables. Then, the optimal number of clusters was determined based on the value most frequently suggested by thirty different validation indices. Subsequently, both K-means and hierarchical clustering were performed using the selected number of clusters, and their performance was compared using the Silhouette coefficient and the DBI, CHI and Dunn indices. The final step consisted of exploring the cluster composition and interpreting the resulting groups.

### 2.3. Statistical Analysis and Cluster Exploration

Once the algorithm showing the best performance indices for each trial was identified, cluster validation was performed. Specifically, the validation aimed to assess whether the mean values of each feature differed significantly among the identified clusters. To account for repeated measurements within animals, a repeated-measures analysis of variance was conducted using mixed-effects models, with the animal included as a random effect. When more than two clusters were identified, pairwise comparisons between clusters were performed using the Sidak adjustment for multiple testing. Differences were considered statistically significant at *p* < 0.05.

## 3. Results

Table 1 summarizes the clustering performance of the K-means and hierarchical algorithms across the different datasets. K-means outperformed hierarchical clustering in terms of the average silhouette, DBI and CHI, yielding higher average silhouette scores (0.17–0.18 vs. 0.10–0.12 for K-means and hierarchical, respectively), higher CHI values (1034–3877 vs. 729–2109 for K-means and hierarchical, respectively), and lower DBI values (2.05–2.16 vs. 2.11–2.5 for K-means and hierarchical, respectively). Conversely, the Dunn Index showed better results for the hierarchical algorithm, with values ranging from 0.03 to 0.05.

**Figure 3:**
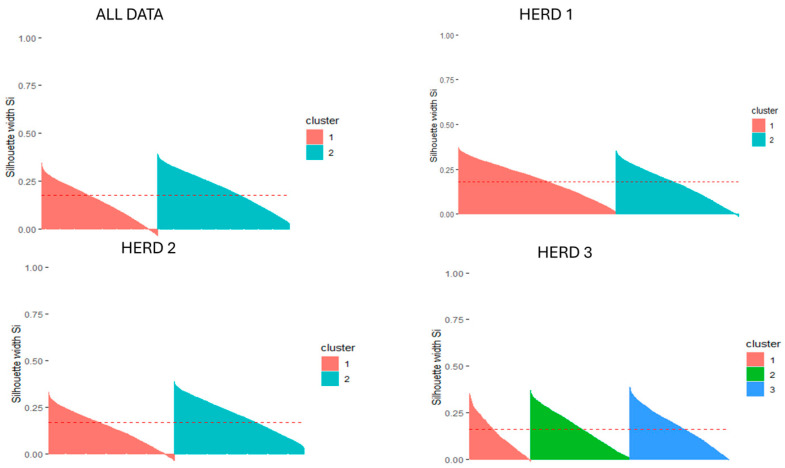
Silhouette analysis obtained after performing the K-means algorithm for the combined dataset and for herds 1, 2 and 3. The red dashed line is the average silhouette.

Based on these results, K-means was selected as the most appropriate unsupervised learning algorithm for this study, as three out of four indices indicated superior performance. Figure 3 provided a graphical representation of the silhouette analysis for the clusters generated by the K-means algorithm, both for the combined dataset and for each individual herd. The silhouette analysis revealed an overall coherent cluster structure, with only a small proportion of observations exhibiting negative silhouette values, indicative of potential misclassifications.

In the combined dataset, 3.63% of the observations were identified as misclassified, mainly concentrated in Cluster 1, suggesting that this cluster was less defined than the others. This interpretation was supported by the lower average silhouette value for Cluster 1 (0.14) compared with Cluster 2 (0.21). At the herd level, for Herd 1 Cluster 1 had no misclassified observation, while Cluster 2 contained 65 misclassified observations (1.13%). In Herd 2, only Cluster 1 showed a misclassification rate of 4%, corresponding to 223 observations. Finally, in Herd 3, Cluster 1 included 35 misclassified cases (2.75%), whereas Cluster 2 included only 13 observations (0.63%).

Table 2 reports the mean ± standard deviation of each variable for the clusters identified by the K-means algorithm applied to the combined dataset. The latter comprised 16,702 test-day records from 1341 buffaloes. Table 3, Table 4 and Table 5 present the corresponding results for Herds 1, 2, and 3, respectively, based on separate K-means analyses carried out within each herd. In Table 2, referring to the combined dataset analysis, Cluster 1 included 7825 observations (46%), whereas Cluster 2 contained 8877 observations (54%). Most of the features showed statistically significant differences between clusters (*p* < 0.001).

Herd 1 included 493 animals and 5728 test-day records. The optimal number of clusters was two. Cluster 1 comprised 2505 observations (43%), whereas Cluster 2 included 3223 observations (57%). The corresponding results are reported in Table 3. All features showed statistically significant differences between clusters (*p* < 0.001), except parity order and age at first calving.

Herd 2 included 421 animals, and the clustering analysis was performed on 5574 observations. Cluster 1 comprised 2741 observations (49%), whereas Cluster 2 contained 2833 observations (51%). The corresponding results are reported in Table 4. All features showed statistically significant differences between clusters, except parity order and age at first calving.

Herd 3 consisted of 427 animals and 5400 test-day records. Cluster 1 comprised 1270 observations (23%), Cluster 2 included 2067 (38.6%), and Cluster 3 contained 2063 (38.4%). The corresponding results are reported in Table 5. Many features showed statistically significant differences (*p* < 0.001) among the three clusters, whereas daily milk yield did not differ significantly between Clusters 1 and 2 while lactation milk yield did not differ significantly between Clusters 2 and 3. Supplementary Materials (Figure S2) supported the evidence presented in Table 2, Table 3, Table 4 and Table 5.

## 4. Discussion

The main novelty of this study relied on the application of unsupervised ML algorithms to buffalo breeding, using routine test-day records. Unsupervised learning approach was chosen because the objective of the work was to uncover natural patterns and groupings within the data, without relying on predefined categories or labels. This methodology made it possible to identify latent structures that improve the understanding of production dynamics and support data-driven decision-making in herd management [21]. Unsupervised algorithms have previously been applied to a range of livestock datasets. For example, Lee et al. [22] performed k-medoids clustering on Holstein cows based on lactation curve characteristics, identifying six groups that included four typical and two atypical lactation curve shapes. Their model highlighted physiological differences among clusters, suggesting possible implications for individual cow management. Moreover, Brotzman et al. [14] successfully applied PCA and hierarchical algorithm to differentiate dairy farms in the US according to productivity and breeding systems. Finally, PCA followed by a hierarchical clustering algorithm has been applied to assess Australian dairy farmers’ preferences for cow trait improvements [23]. Farmers’ preferences were collected through a survey in which each participant was asked to indicate the traits they considered most important. These findings were used to guide trait and index selection.

### 4.1. Clustering Performance

To identify patterns in buffalo characteristics and test-day records, both K-means and hierarchical clustering algorithms were applied to the combined dataset, as well as to the three individual herds. This double approach enabled the evaluation of algorithm’s robustness under different herd conditions. Overall, K-means consistently outperformed hierarchical clustering, as evidenced by the average silhouette and CHI values, and DBI values. For K-means, silhouette scores ranged from 0.17 to 0.18, indicating limited cohesion within clusters. These results suggested that intra-cluster similarity was only slightly greater than inter-cluster similarity [24], implying that many data points were positioned near cluster boundaries. Approximately 4% of the observations displayed negative silhouette values, indicating potential misclassifications. Although most points had positive scores, many were close to zero, further highlighting the blurred boundaries, particularly among clusters representing animals in mid-lactation. Similarly, DBI values ranged from 2.05 to 2.16, and CHI values confirmed modest clustering performance. These results indicated the presence of overlapping or weakly defined clusters [19]. It is worth noting that both metrics can be influenced by factors such as data noise, skewed distributions, internal substructures and differences in cluster density [20], which may also have affected clustering performance in this study. The structure and distribution of the data further contribute to these results. Specifically, the data points formed a dense, nearly circular distribution, leading to poor separation between clusters and therefore lower values for separation-based indices. Conversely, the clusters exhibited relatively high internal cohesion. Despite these limitations, the clusters generated by K-means were meaningful from a domain-specific standpoint and provided valuable insights into the variability of animal characteristics and production traits.

### 4.2. Clustering Ability to Create Homogenous Groups

Overall, DIM was useful for clustering purposes, effectively grouping animals into two or three distinct lactation phases. Moreover, in the combined dataset, the K-means algorithm separated observations according to milk yield, confirming this trait as one of the most influential in distinguishing clusters. Cluster 1 comprised buffaloes in late lactation, with an average of 213 DIM, having the lowest daily milk yield (6.18 kg) but the highest fat (9.74%) and protein (4.99%) contents. The same cluster also showed the highest SCS (4.16), a relatively long lactation length (324 days), and a total lactation milk yield of 2576 kg. In contrast, Cluster 2 included animals in early lactation (average 89 DIM), characterized by the highest daily milk yield (10.89 kg) and the lowest fat (7.47%) and protein (4.53%) contents, consistent with the expected dilution effect near the peak lactation [25]. This cluster also exhibited the lowest SCS (2.99), suggesting better udder health. A comparable clustering pattern was observed in Herds 1 and 2, where two distinct clusters emerged. In Herd 1, Cluster 1 consisted of late-lactation animals (average 211 DIM), showing the highest fat (9.94%) and protein (4.90%) contents, the lowest daily milk yield (9.44 kg), and the highest SCS (3.96), reflecting declining productivity and potential deterioration of udder health. The higher SCS in late-lactation animals may indicate cumulative stress or subclinical mastitis, as previously reported in previous research [26]. Cluster 2 grouped animals in early lactation (86 DIM) that showed higher daily milk yield (12.67 kg) and lower fat (7.23%) and protein (4.43%) contents.

Concerning Herd 2, the pattern was similar: K-means successfully clustered animals into two main lactation stages, grouping buffaloes that exhibited high daily milk yield and lactose content with lower fat and protein content. SCS also followed this trend, increasing in the late lactation cluster.

Consistent with clustering analysis, the ANOVA results showed significant differences among clusters for most of the evaluated features. In Herds 1 and 2, similar trends were observed, except for parity order and age at first calving, which did not differ significantly. A possible explanation is that these variables contributed less to cluster discrimination.

In Herd 3, the K-means analysis identified three distinct clusters, consistently separating animals according to lactation stage. In this case, lactation was divided into early, mid-, and late stages based on DIM. As in the other herds, animals in each lactation stage exhibited different milk production patterns. This result clearly reflected the typical lactation curve [27], where milk yield increased progressively during early lactation (86 DIM; 9.95 kg), remained stable during mid-lactation (122 DIM; 10.07 kg), and gradually declined in late lactation (219 DIM; 6.18 kg). The early-lactation cluster also showed higher lactose concentrations, consistent with the osmotic role of lactose in milk secretion [28]. Furthermore, an inverse relationship between lactose and SCS was observed, confirming previous findings [29]. Interestingly, Cluster 1 grouped older animals compared with Clusters 2 and 3 (5.82, 3.69, and 3.69 years, respectively), and these animals also exhibited the highest total lactation milk yield. This association aligned with physiological expectations, as the genetic potential for milk production is typically fully expressed from the second lactation onward [30]. The ANOVA for Herd 3 revealed statistically significant differences among clusters for almost all traits.

### 4.3. Practical Application

The results obtained with the K-means algorithm were consistent with a recent study [31], which demonstrated the effectiveness of clustering techniques in capturing meaningful variation in dairy production traits. K-means successfully differentiated physiological stages based on milk yield and composition in dairy cows, with cluster separation typically occurring after the lactation peak, between 70 and 90 DIM [32]. Despite this, it is important to highlight that the results obtained are valid for the Italian Mediterranean buffaloes, which are the only breed included in the dataset. Other buffalo breeds may exhibit different productive and reproductive performances, and therefore the results could differ [33]. However, since the data used in the present analysis is readily available and commonly recorded in the livestock sector, the proposed methodology could also be applied to other breeding systems and species. In a study by Trapanese et al. [34] a similar approach was applied to Saanen and Camosciata goat breeds, where, as in the present study, DIM strongly influenced cluster formation. These findings suggested that the proposed approach can provide valuable insights not only for Italian buffalo breeding but also for other livestock species. From a practical perspective, understanding the physiology of lactation is essential for evaluating herd genetic potential, managing reproductive and nutritional strategies and optimizing overall milk yield. Clustering analysis offers a data-driven framework for grouping animals according to physiological and production parameters rather than relying solely on farmer experience or intuition. This approach is particularly relevant to nutritional management [35]. It is well established that animals at different DIM have distinct energy and nutrient requirements, which should be addressed using precision feeding strategies. Our findings indicate that K-means clustering can support more precise and objective animal grouping based on routinely collected data. Moreover, according to Kalantari et al. [36], data-driven grouping strategies can improve income over feed costs by simultaneously increasing milk output and reducing unnecessary feed expenditures, resulting in a significant positive economic impact.

## 5. Conclusions

This study proposed an automated approach to exploring routine buffalo milk data, offering a potential tool to support and enhance herd management practices. Although the evaluation indices were suboptimal, the K-means clustering produced coherent groupings that reflected the physiological patterns of lactation. One of the main advantages of this method lies in the accessibility of the data, which are routinely collected by farmers, technicians and automated systems, thereby eliminating the need for additional data collection efforts. The findings suggested that clustering techniques, particularly K-means, can serve as a foundation for modernizing buffalo breeding strategies. By grouping buffaloes according to lactation stage and performance profiles, it may be possible to optimize nutritional plans, improve resource allocation and enhance overall productivity. Looking ahead, this methodology could be further refined by integrating detailed information on milk protein and lipid fractions in relation to feeding regimes. Such integration would enable a more precise characterization of milk composition dynamics across lactation stages and nutritional contexts, ultimately contributing to the design of diet plans tailored to maximize both milk quality and animal health. Finally, the recent adoption of automatic milking systems could serve as a continuous data source, providing daily milk records. Such detailed and frequent data could improve clustering performance and allow the use of more advanced or alternative clustering algorithms.

## Figures and Tables

**Table 1:** Comparison of clustering performance ^1^ between hierarchical and K-means clustering algorithms applied to the complete dataset and three individual herds.

Dataset	K-Means					Hierarchical				
	N of Clusters	Average Silhouette Score	DBI	CHI	Dunn	N of Clusters	Average Silhouette Score	DBI	CHI	Dunn
Total dataset	2	0.18	2.07	3877	0.0213	3	0.11	2.30	2109	0.0300
Herd 1	2	0.18	2.08	1307	0.0272	3	0.11	2.50	992	0.0343
Herd 2	2	0.17	2.16	1191	0.0298	3	0.10	2.29	729	0.0534
Herd 3	3	0.18	2.05	1034	0.0241	3	0.12	2.11	951	0.0451

^1^ Clustering performance: Silhouette score: indicates how well each observation fits within its cluster; DBI: measures cluster compactness and separation. CHI: evaluates the ratio of between-cluster dispersion to within-cluster dispersion, Dunn: assesses the minimum inter-cluster distance relative to the maximum intra-cluster distance.

**Table 2:** Animal production traits, reproductive performance and milk quality for each cluster identified by the K-means clustering applied on the combined dataset. Data were shown as means ± standard deviation. NS = not significant (*p* > 0.05).

Traits	Cluster 1	Cluster 2	*p*-Value
N. of samples	7825	8877	
Animal characteristics:			
Parity order	1.58 ± 0.77	1.68 ± 0.85	<0.0001
Primiparous (%)	52.65	55.98	
Days in milk (d)	213 ± 70	89 ± 55	<0.0001
Lactation length (d)	324 ± 75	293 ± 60	<0.0001
Age at first calving (mo)	33 ± 5	34 ± 4	NS
Age (y)	4.18 ± 1.13	3.95 ± 1.17	<0.0001
Milk production and composition:			
Lactation milk yield (kg)	2476 ± 758	2638 ± 736	<0.001
Daily milk yield (kg/d)	6.18 ± 2.40	10.89 ± 3.00	<0.0001
Fat (%)	9.74 ± 1.18	7.47 ± 1.05	<0.0001
Protein (%)	4.99 ± 0.36	4.53 ± 0.33	<0.0001
Fat: Protein	1.96 ± 0.22	1.65 ± 0.23	<0.0001
Lactose (%)	4.58 ± 0.37	4.86 ± 0.27	<0.0001
Somatic cell score	4.16 ± 1.57	2.99 ± 1.54	<0.0001

**Table 3:** Animal production traits, reproductive performance and milk quality for each cluster identified by K-means clustering applied on Herd 1. Data were shown as means ± standard deviation. NS = not significant (*p* > 0.05).

Traits	Cluster 1	Cluster 2	*p*-Value
N. of samples	2505	3223	
Animal characteristics:			
Parity order	1.61 ± 0.77	1.59 ± 0.78	NS
Primiparous (%)	55.96	55.83	
Days in milk (d)	211.31 ± 61.57	86.94 ± 50.77	<0.0001
Lactation length (d)	302.33 ± 59.42	283.57 ± 52.48	<0.0001
Age at first calving (mo)	34.72 ± 2.91	34.67 ± 2.68	NS
Age (y)	4.20 ± 1.05	3.75 ± 0.96	<0.0001
Milk production and composition:			
Lactation Milk yield (kg)	3034 ± 692	3024 ± 655	<0.001
Daily milk yield (kg/d)	8.19 ± 2.40	12.67 ± 2.75	<0.0001
Fat (%)	9.44 ± 1.10	7.23 ± 1.03	<0.0001
Protein (%)	4.90 ± 0.37	4.43 ± 0.32	<0.0001
Fat: Protein	1.93 ± 0.20	1.64 ± 0.23	<0.0001
Lactose (%)	4.64 ± 0.27	4.85 ± 0.27	<0.0001
Somatic cell score	3.96 ± 1.33	3.08 ± 1.51	<0.0001

**Table 4:** Animal production traits, reproductive performance and milk quality for each cluster identified by K-means clustering applied on Herd 2. Data were shown as means ± standard deviation. NS = not significant (*p* > 0.05).

Traits	Cluster 1	Cluster 2	*p*-Value
Number of samples	2741	2833	
Animal characteristics:			
Parity order	1.55 ± 0.78	1.57 ± 0.80	NS
Primiparous (%)	57.03	56.49	
Days in milk (d)	229 ± 72	86.00 ± 54.63	<0.0001
Lactation length (d)	352.90 ± 90.94	314.76 ± 83.22	<0.0001
Age at first calving (mo)	32.10 ± 6.48	31.26 ± 4.83	NS
Age (y)	4.05 ± 1.22	3.60 ± 1.19	<0.0001
Milk production and composition:			
Lactation Milk yield (kg)	2214 ± 718	2107 ± 733	<0.0001
Daily milk yield (kg/d)	4.78 ± 2.06	8.64 ± 2.80	<0.0001
Fat (%)	10.08 ± 1.25	8.02 ± 1.10	<0.0001
Protein (%)	5.02 ± 0.36	4.71 ± 0.31	<0.0001
Fat: Protein	2.01 ± 0.23	1.71 ± 0.24	<0.0001
Lactose (%)	4.49 ± 0.48	4.87 ± 0.27	<0.0001
Somatic cell score	4.77 ± 1.62	3.51 ± 1.59	<0.0001

**Table 5:** Animal production traits, reproductive performance and milk quality for each cluster identified by K-means clustering applied on Herd 3. Data were shown as means ± standard deviation. Means not sharing a common letter are significantly different at *p* < 0.05. NS = not significant (*p* > 0.05).

Traits	Cluster 1	Cluster 2	Cluster 3	*p*-Value
Number of samples	1270	2067	2063	
Animal characteristics:				
Parity order	2.89 ± 0.65 ^A^	1.31 ± 0.48 ^C^	1.45 ± 0.60 ^B^	<0.0001
Primiparous (%)	0	60.56	56.74	
Days in milk (d)	122.37 ± 64.22 ^B^	84.04 ± 53.62 ^C^	219.95 ± 59.30 ^A^	<0.001
Lactation length (d)	270.83 ± 37.84 ^C^	303.91 ± 48.30 ^B^	311.77 ± 49.80 ^A^	<0.0001
Age at first calving (mo)	35.78 ± 3.39	37.17 ± 3.72	36.64 ± 3.57	NS
Age (y)	5.82 ± 0.82 ^A^	3.69 ± 0.73 ^C^	3.69 ± 0.73 ^B^	<0.001
Milk production and composition:				
Lactation Milk yield (kg)	2556 ± 610 ^A^	2480 ± 539 ^B^	2441 ± 548 ^B^	<0.0001
Daily milk yield (kg/d)	10.07 ± 3.06 ^A^	9.95 ± 2.44 ^A^	6.18 ± 1.97 ^B^	<0.0001
Fat (%)	8.06 ± 1.15 ^B^	7.25 ± 1.01 ^C^	9.70 ± 1.16 ^A^	<0.0001
Protein (%)	4.59 ± 0.35 ^B^	4.54 ± 0.33 ^C^	5.04 ± 0.34 ^A^	<0.0001
Fat/Protein	1.76 ± 0.23 ^B^	1.60 ± 0.22 ^C^	1.93 ± 0.21 ^A^	<0.0001
Lactose (%)	4.76 ± 0.31 ^B^	4.87 ± 0.26 ^A^	4.58 ± 0.28 ^C^	<0.0001
Somatic cell score	3.18 ± 1.42 ^B^	2.36 ± 1.46 ^C^	3.55 ± 1.47 ^A^	<0.0001

## Data Availability

Data are contained within the article and Supplementary Materials.

## Supplementary Materials

The following supporting information can be downloaded at: https://www.mdpi.com/article/10.3390/ani15223246/s1, Figure S1: Correlation matrix of the combined dataset. Figure S2: Density plot of Days in Milk among clusters.

## References

[1] Gong L., Luo L., Gao J., Xiong Y., Chen C., Gan H., Song H., Morrone S., Dimauro C., Gambella F. (2022). Industry 4.0 and Precision Livestock Farming (PLF): An up to Date Overview across Animal Productions. Sensors.

[2] Peyraud J.-L., MacLeod M. (2020). Future of EU Livestock: How to Contribute to a Sustainable Agricultural Sector?.

[3] Trapanese L., Bifulco G., Calanni Macchio A., Aragona F., Purrone S., Campanile G., Salzano A. (2025). Precision livestock farming applied to the dairy sector: 50 years of history with a text mining and topic analysis approach. Smart Agric. Technol..

[4] Pugliese R., Regondi S., Marini R. (2021). Machine learning-based approach: Global trends, research directions, and regulatory standpoints. Data Sci. Manag..

[5] Le Duc T., Leiva R.G., Casari P., Östberg P.O. (2019). Machine learning methods for reliable resource provisioning in edge-cloud computing: A survey. ACM Comput. Surv..

[6] Can Machine Learning Algorithms Perform Better than Multiple Linear Regression in Predicting Nitrogen Excretion from Lactating Dairy Cows|Scientific Reports. https://www.nature.com/articles/s41598-022-16490-y.

[7] Wang J., Bell M., Liu X., Liu G. (2020). Machine-Learning Techniques Can Enhance Dairy Cow Estrus Detection Using Location and Acceleration Data. Animals.

[8] Tremblay M., Kammer M., Lange H., Plattner S., Baumgartner C., Stegeman J.A., Duda J., Mansfeld R., Döpfer D. (2018). Identifying poor metabolic adaptation during early lactation in dairy cows using cluster analysis. J. Dairy Sci..

[9] Franceschini S., Grelet C., Leblois J., Gengler N., Soyeurt H. (2022). Can unsupervised learning methods applied to milk recording big data provide new insights into dairy cow health?. J. Dairy Sci..

[10] Matera R., Pierro F., Santinello M., Iraci Fuintino A., Pacelli G., Norton T., Neglia G. (2025). Precision livestock farming in buffalo species: A sustainable approach for the future. Smart Agric. Technol..

[11] Warner D., Vasseur E., Lefebvre D.M., Lacroix R. (2020). A machine learning based decision aid for lameness in dairy herds using farm-based records. Comput. Electron. Agric..

[12] Costa A., Negrini R., De Marchi M., Campanile G., Neglia G. (2020). Phenotypic Characterization of Milk Yield and Quality Traits in a Large Population of Water Buffaloes. Animals.

[13] Ali A.K.A., Shook G.E. (1980). An Optimum Transformation for Somatic Cell Concentration in Milk1. J. Dairy Sci..

[14] Brotzman R.L., Cook N.B., Nordlund K., Bennett T.B., Gomez Rivas A., Döpfer D. (2015). Cluster analysis of Dairy Herd Improvement data to discover trends in performance characteristics in large Upper Midwest dairy herds. J. Dairy Sci..

[15] Abreu B.D.S., Barbosa S.B.P., Silva E.C.D., Santoro K.R., Batista Â.M.V., Martinez R.L.V. (2020). Principal component and cluster analyses to evaluate production and milk quality traits. Rev. Ciência Agronômica.

[16] Çelik M., Dadaşer-Çelik F., Dokuz A.Ş. Anomaly detection in temperature data using DBSCAN algorithm. Proceedings of the 2011 International Symposium on Innovations in Intelligent Systems and Applications.

[17] Atif M., Farooq M., Abiad M., Shafiq M. (2024). The least sample size essential for detecting changes in clustering solutions of streaming datasets. PLoS ONE.

[18] Charrad M., Ghazzali N., Boiteau V., Niknafs A. (2014). NbClust: An R Package for Determining the Relevant Number of Clusters in a Data Set. J. Stat. Softw..

[19] Arbelaitz O., Gurrutxaga I., Muguerza J., Pérez J.M., Perona I. (2013). An extensive comparative study of cluster validity indices. Pattern Recognit..

[20] Davies D.L., Bouldin D.W. (1979). A Cluster Separation Measure. IEEE Trans. Pattern Anal. Mach. Intell..

[21] Wong P.C., von Davier A.A., Mislevy R.J., Hao J. (2021). Unsupervised Machine Learning. Computational Psychometrics: New Methodologies for a New Generation of Digital Learning and Assessment: With Examples in R and Python.

[22] Lee M., Lee S., Park J., Seo S. (2020). Clustering and Characterization of the Lactation Curves of Dairy Cows Using K-Medoids Clustering Algorithm. Animals.

[23] Martin-Collado D., Byrne T.J., Amer P.R., Santos B.F.S., Axford M., Pryce J.E. (2015). Analyzing the heterogeneity of farmers’ preferences for improvements in dairy cow traits using farmer typologies. J. Dairy Sci..

[24] Khan I.K., Daud H.B., Zainuddin N.B., Sokkalingam R., Farooq M., Baig M.E., Ayub G., Zafar M. (2024). Determining the optimal number of clusters by Enhanced Gap Statistic in K-mean algorithm. Egypt. Inform. J..

[25] Silvestre A.M., Martins A.M., Santos V.A., Ginja M.M., Colaço J.A. (2009). Lactation curves for milk, fat and protein in dairy cows: A full approach. Livest. Sci..

[26] Kirsanova E., Heringstad B., Lewandowska-Sabat A., Olsaker I. (2019). Alternative subclinical mastitis traits for genetic evaluation in dairy cattle. J. Dairy Sci..

[27] Luna-Palomera C., Domínguez-Viveros J., Aguilar-Palma G.N., Castillo-Rangel F., Sánchez-Dávila F., Macías-Cruz U., Luna-Palomera C., Domínguez-Viveros J., Aguilar-Palma G.N., Castillo-Rangel F. (2021). Analysis of the Lactation Curve of Murrah Buffaloes with Mixed Non-Linear Models. Chil. J. Agric. Anim. Sci..

[28] Gargiulo J.I., Garcia S.C., Hovey R.C. (2025). Sources of variation underlying the production of lactose by dairy cows. J. Dairy Sci..

[29] Alessio D., Velho J., Mcmanus C., Knob D., Vancin F., Antunes G., Busanello M., Carli F., Thaler-Neto A. (2021). Lactose and its relationship with other milk constituents, somatic cell count, and total bacterial count. Livest. Sci..

[30] Cattaneo L., Piccioli-Cappelli F., Minuti A., Trevisi E. (2023). Metabolic and physiological adaptations to first and second lactation in Holstein dairy cows. J. Dairy Sci..

[31] Rebuli K.B., Ozella L., Vanneschi L., Giacobini M. (2023). Multi-algorithm clustering analysis for characterizing cow productivity on automatic milking systems over lactation periods. Comput. Electron. Agric..

[32] Ghavi Hossein-Zadeh N. (2016). Comparison of non-linear models to describe the lactation curves for milk yield and composition in buffaloes (*Bubalus bubalis*). Animal.

[33] Minervino A.H.H., Zava M., Vecchio D., Borghese A. (2020). *Bubalus bubalis*: A Short Story. Front. Vet. Sci..

[34] Trapanese L., Bifulco G., Aragona F., GianMaria P., Pedota G., Pasquino N., Salzano A. Explorative analysis of Saanen and Camosciata goats data through an unsupervised machine learning approach. Proceedings of the 2024 International Workshop Measurements and Applications Inveterinary and Animal Sciences.

[35] Barrientos-Blanco J.A., White H., Shaver R.D., Cabrera V.E. (2022). Graduate Student Literature Review: Considerations for nutritional grouping in dairy farms. J. Dairy Sci..

[36] Kalantari A.S., Armentano L.E., Shaver R.D., Cabrera V.E. (2016). Economic impact of nutritional grouping in dairy herds. J. Dairy Sci..

