# Supplementary material for: Comparison of K-Means and Hierarchical Clustering Methods for Buffalo Milk Production Data
Source: Animals (Basel). 2025 Nov 9;15(22):3246. doi: 10.3390/ani15223246 (PMC12649153; doi:10.3390/ani15223246)
Supplement: Supplementary file 1 [file animals-15-03246-s001.zip › animals-3923203-supplementary.pdf]

Supplementary materials

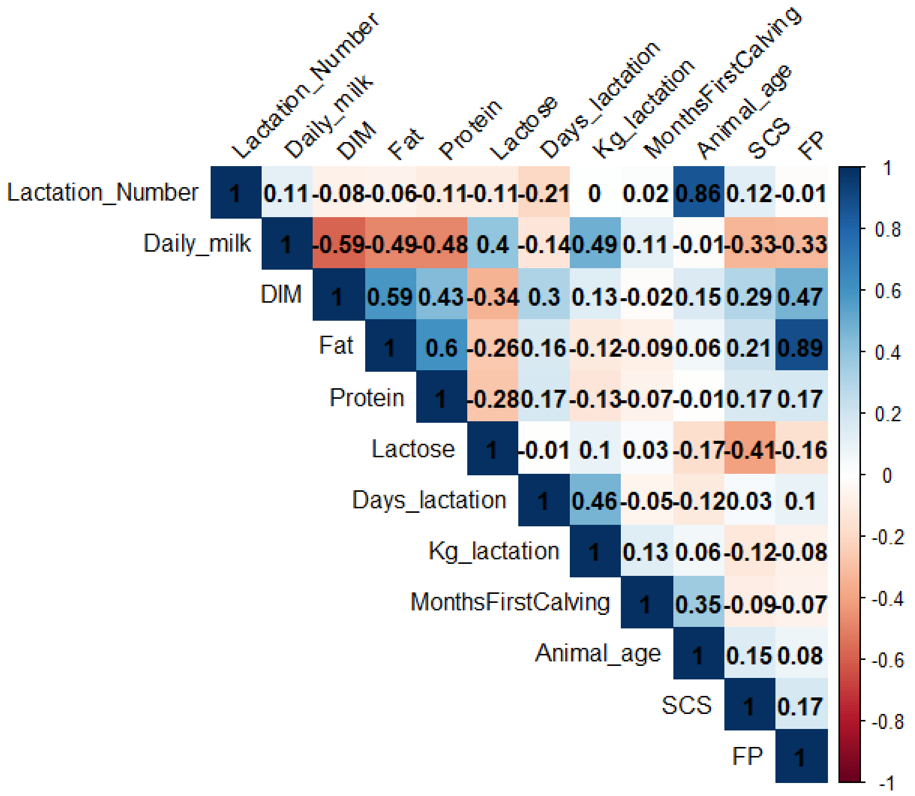

Figure S1.Correlation matrix of the combined dataset.

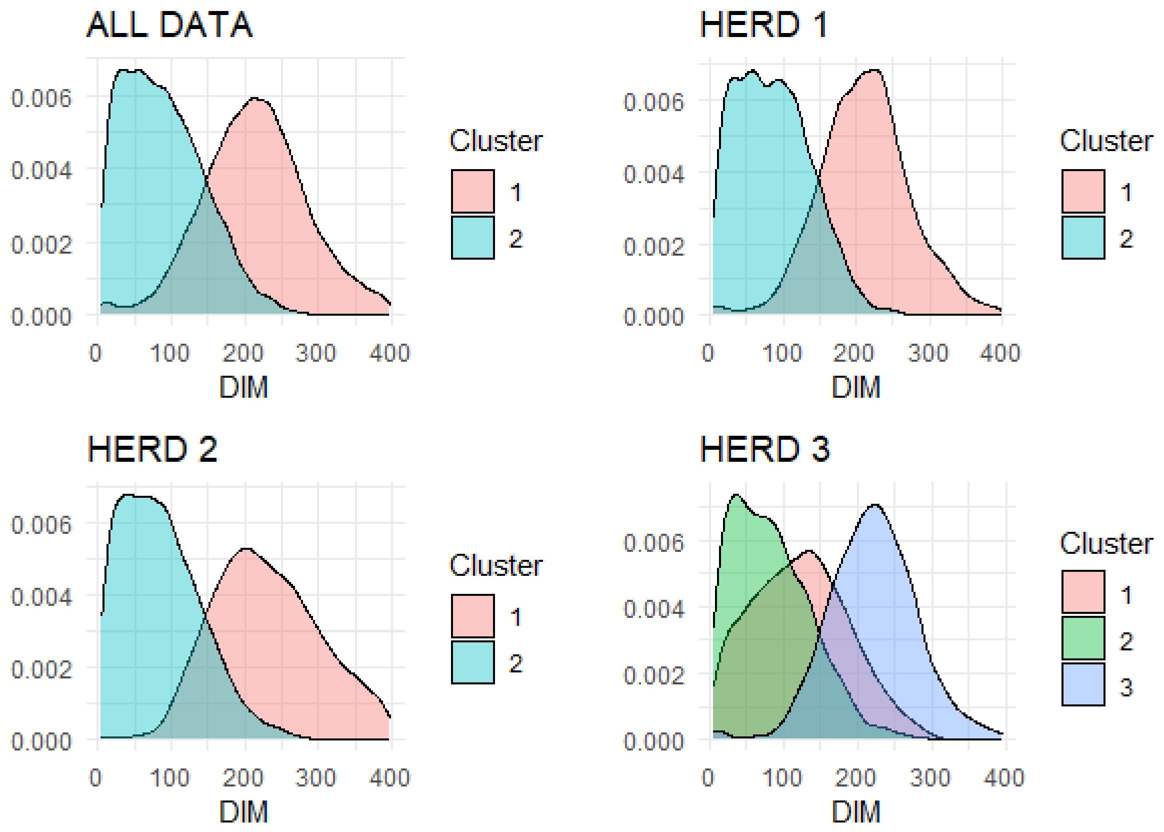

Figure S2. Density distribution of DIM (Days In Milk) among clusters. Each subplot reports the results of one dataset.
